# Immune checkpoint inhibitors in advanced and recurrent/metastatic cervical cancer

**DOI:** 10.3389/fonc.2022.996495

**Published:** 2022-10-06

**Authors:** Xiling Han, Wei-wei Chang, Xiaoping Xia

**Affiliations:** ^1^ Department of Obstetrics and Gynecology, Anhui Provincial Children’s Hospital, Children’s Hospital of Fudan University Anhui Hospital, Children’s Hospital of Anhui Medical University, Hefei, China; ^2^ Department of Epidemiology and Health Statistics, School of Public Health, Wannan Medical College, Wuhu, China

**Keywords:** immune checkpoint inhibitors, cervical cancer, immunotherapy, vaginal microecology, intestinal microecology

## Abstract

Cervical cancer (CC) poses a serious threat to women’s health. Although many early-stage patients have a good prognosis, there are still a lack of effective therapies for advanced and recurrent/metastatic CC. In this context, immunotherapy and immune checkpoint inhibitors (ICIs) are particularly likely to play a role in the treatment of cervical tumors in a variety of disease settings. Some promising immune checkpoints include programmed cell death 1 (PD-1), programmed death ligand 1 (PD-L1) and cytotoxic T lymphocyte antigen 4 (CTLA-4), which exert immunomodulatory effects as negative regulators of T-cell activation and suppress immune responses in cervical cancer through cancer cell immune evasion. Initial trials of ICIs for CC have shown encouraging results in terms of objective response rate (ORR), progression-free survival (PFS), and overall survival (OS), both monotherapy and combination strategies. Meanwhile, human papillomavirus, vaginal microecology and intestinal microenvironment play an important role in CC, which provides new treatment directions. This review analyzed a number of completed or ongoing clinical trials of ICIs in the treatment of advanced and recurrent/metastatic CC. And we also analyzed the important relationship between vaginal microecology and intestinal microecology with CC and their related immunotherapy prospects.

## 1 Introduction

A gynecological malignancy most commonly found in women is cervical cancer (CC), which poses a serious threat to women’s health, with approximately 570,000 women diagnosed each year, resulting in approximately 311,000 deaths, especially in developing countries (1–3). Due to a lack of cytological screening and/or high-risk human papilloma virus (HR-HPV) DNA testing, as well as the difficulty of vaccinating every woman against HPV. More than 70% of CC cases diagnosed in developing countries are locally invasive or metastatic, resulting in a high death rate (4). CC is the fourth-leading cause of cancer-related death worldwide (5).

HPV infection includes both low-risk and high-risk types, of which high-risk HPV are the main factors of cervical intraepithelial neoplasia (CIN) and CC. After a definitive diagnosis, the standard treatment regimen is determined based on the patient’s stage of disease and clinicopathologic risk factors, which may include surgery and a combination of chemotherapy and radiation (6). According to the Federation of Gynecology and Obstetrics (FIGO), recurrence rates between 11% and 22% in patients with stage IB-IIA CC and between 28% and 64% for patients with stage IIB-IV CC (7, 8). Although widespread use of HPV vaccines and CC screening and new therapies, including bevacizumab and pembrolizumab, have been shown to improve survival, the 5-year overall survival (OS) is only approximately 15% in recurrent, persistent or metastatic CC (9), and other effective novel therapies for advanced and recurrent/metastatic CC remain unmet. Therefore, new treatment modalities and paradigms are needed to improve the prognosis of women diagnosed with CC.

In this context, immunotherapy and immune checkpoint inhibitors (ICIs) are particularly likely to play a role in the treatment of cervical tumors in a variety of disease settings. CC is also known as T-cell inflammatory cancer. Long-term HPV infection is closely related to the development of CC (10) and significantly affects the expression of PD-L1 in tumor tissues, so that ICIs may improve the prognosis of CC associated with HPV infection. For this reason, clinical trials have been conducted to assess the efficacy of ICIs against CC. However, treatment with ICIs is associated with a variety of diverse and distinct immune-related adverse events (irAEs). The most frequent irAEs associated with anti-PD-1 monoclonal antibody (mAb) treatment are thyroid dysfunctions, while hypophysis is mostly linked to anti-CTLA-4 treatment (11). Moreover, HR-HPV infection can exacerbate vaginal microecological disorders, and disturbances in the intestinal microenvironment can lead to a decrease in the body’s immunity. Many studies have confirmed that the two are clearly related to the occurrence and prognosis of CC. This offers new promising treatment insights.

This review summarizes the major ICIs used in the treatment of CC, with a particular focus on the rationale and outcomes of clinical trials. Moreover, the role of new treatment directions, including vaginal microecology and intestinal microecology, as well as applications in the treatment of advanced and recurrent/metastatic CC were discussed.

## 2 Immune checkpoint inhibitors and cervical cancer

### 2.1 The mechanism of cervical cancer

Persistent infection with high-risk human papilloma virus (HR-HPV) is an important condition for the occurrence of CC. Meanwhile, the infection and self-clearing mechanism of HR-HPV is related to the immune function of the host. In clinical treatment, improving the immunity of patients while treating viral infections can inhibit HR-HPV to a certain extent and effectively prevent the occurrence of CC.

T lymphocytes are the main effector cells of the body’s cellular immunity. CD4+ T cells and CD8+ T cells play a key role in local and systemic tumor immune regulation. When the body is stimulated by viral antigens, CD4 + T cells are activated and differentiated, producing Th1 cells and Th2 cells. In HPV-infected CC patients, we found that the expression of CD4+ T cells was decreased, the expression of Th1 and Th2 cytokines was abnormal. Besides, IL-2, IFN-γ, TNF-α, IL-4, IL-6, IL-10, etc., were all overexpressed, and Th2 cytokines were actively expressed in the balance of Th1/Th2, which promoted the occurrence and progression of tumors. In addition, the E6 and E7 oncogenes in HPV can bind to the P53 tumor suppressor gene, promote the degradation of the P53 gene, abrogate the regulation of the cell cycle and cause it to proliferate indefinitely. HR-HPV can also regulate minichromosomal maintenance proteins and cell division cycle protein-6, which makes cells differentiate into heterolytic cells (12, 13). For patients with persistent HR-HPV infection, HPV acts on the body’s immune system after infection by downregulating MHC, affecting viral antigen presentation and inhibiting the activation of cytotoxic T cells and NK cells. HPV E5 protein can escape the immune surveillance of CD4+ T cells and CD8+ T cells and affect the immune barrier of the body, which leads to the occurrence of CC.

### 2.2 Immune checkpoint inhibitors

**Figure 1:**
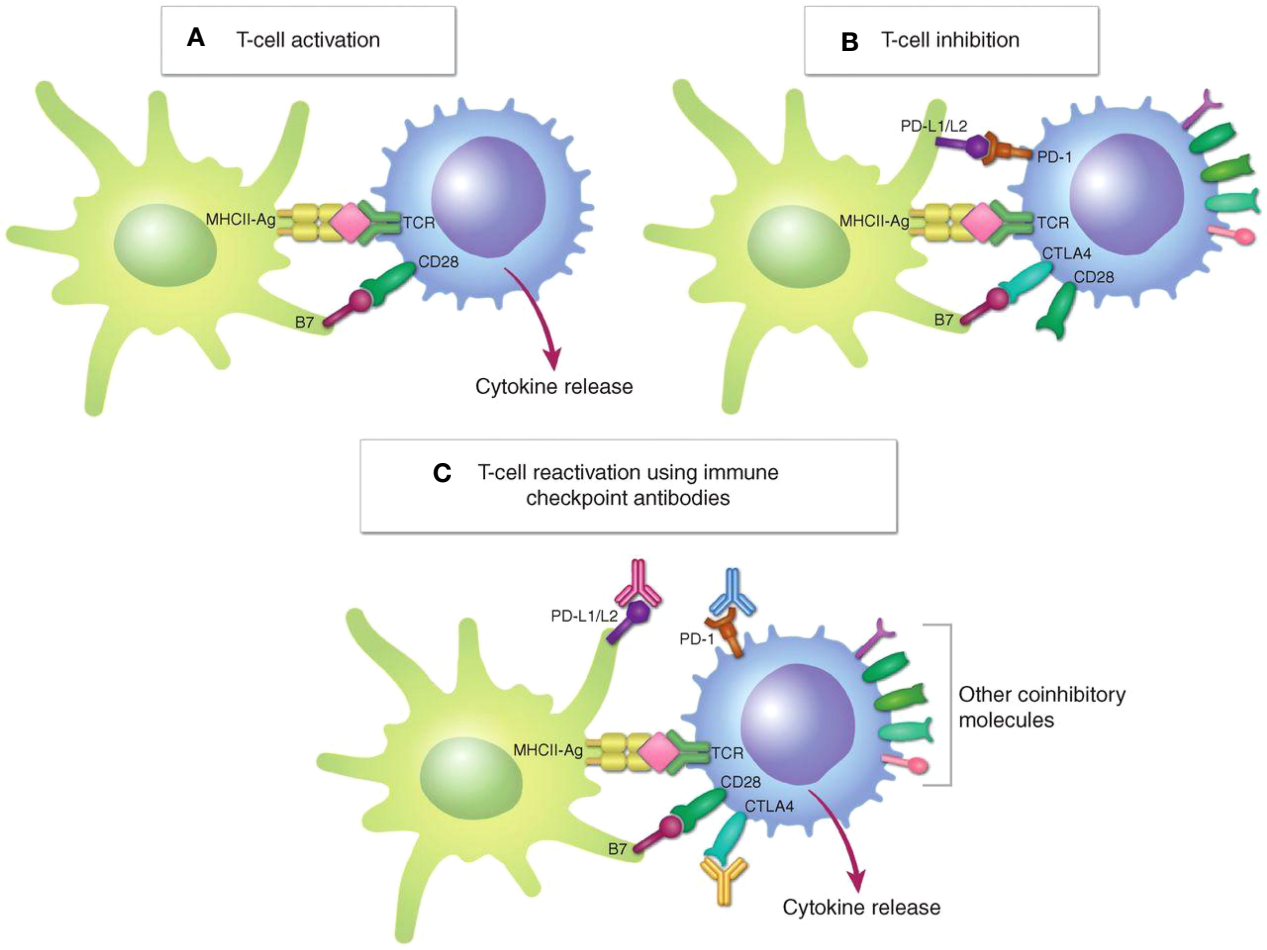
Regulation of T-cell activation. **(A)**, T-cell activation requires both signal 1, TCR engagement with the MHC–peptide antigen complex (MHC-Ag) on an APC or a target cell, and signal 2, interaction of the costimulatory receptor CD28 on the T cell with costimulatory B7 molecules (CD80/CD86). **(B)**, In response to T-cell activation, the immune checkpoints CTLA4 and PD-1 are upregulated on the T cell and bind to B7 and PD-L1/L2, respectively, to inhibit T-cell activation. **(C)**, Immune checkpoint antibodies targeting CTLA4 or PD-1/PD-L1 block these inhibitory interactions, reactivating T cells (14). *Copyright ^©^ 2021, American Association for Cancer Research*.

Activation of antitumor T-cell responses requires costimulatory ligand-receptor interactions (e.g., B7-CD28 interactions) and presentation of immunogenic peptide antigens by the major histocompatibility complex (MHC). Multiple inhibitory mechanisms exist, both intracellular and extracellular, mediated by receptors cytotoxic T-lymphocyte protein 4 (CTLA4) and PD-1, as well as other inhibitory signals—are involved in the inactivation of tumor-infiltrating T cells (**Figure 1** **) (** 14). Immune checkpoint inhibitors (ICIs), which act by blocking the binding of checkpoint proteins to their partner proteins, have shown promise in advanced and recurrent/metastatic cancers such as breast cancer, gynecologic malignancy, cholangiocarcinoma and colorectal cancer (15, 16). Immune checkpoints are immune regulatory molecules that play an important role in evading immunosurveillance. They include programmed cell death 1 (PD-1) and its ligands programmed death ligand 1 (PD-L1), cytotoxic T lymphocyte antigen-4 (CTLA-4), V-domain Ig suppressor of T-cell activation (VISTA), T-cell immunoglobulin domain and mucin domain 3 (TIM-3), B7 homolog 3 (B7-H3), lymphocyte activation gene-3 (LAG-3), etc. (17, 18). PD-L1 is expressed in a variety of immune cells and tumor cells and could bind to PD-1 on T lymphocytes to inhibit its function. Currently, PD-1/PD-L1 is a major immunotherapeutic target for checkpoint inhibition in various cancers.

In one study, clinical information from cervical intraepithelial neoplasia (CIN) and cervical squamous cell carcinoma (SCC) patients was collected, and immunohistochemistry was used to measure the expression of PD-L1 in tumor cells and PD-1 in tumor-associated macrophages (TAMs) and tumor-infiltrating lymphocytes (TILs). The expression levels of PD-L1 and PD-1 are positively correlated with CIN progression and tumor metastasis and can be used as clinical prognostic biomarkers to evaluate CIN and CC. Another study in HPV-related head and neck squamous cell carcinoma (HNSCC) demonstrated that HPV infection significantly increased T-cell infiltration, immune effector cell activation and the diversity of T-cell receptors. Notably, HPV positivity correlated with increased immune cytolytic activity and a T-cell-inflamed gene expression profile. This work provided evidence that HPV status can be used to predict the effectiveness of PD-1 inhibitors in HNSCC, which is independent of PD-L1 expression and tumor mutation burden, and is probably results from an inflamed immune microenvironment induced by HPV infection (19). In addition, a series of studies have also demonstrated that PD-L1 and PD-1 are widely expressed in CC tumor cells and stroma, indicating potential therapeutic targets of PD-1/PD-L1 inhibitors (20–22). As negative regulators of T-cell activation, these ICIs play an immunomodulatory role and inhibit the immune response of CC through immune evasion of cancer cells. Therefore, ICIs are highly anticipated as a potential treatment for cervical cancer (23, 24).

### 2.3 Clinical trials

Since the breakthrough of ICIs, numerous preclinical and clinical studies have been carried out to investigate which of these drugs could work in a complementary fashion and thereby render synergistic combinations with ICIs. Many studies are being conducted with novel combinations of checkpoint inhibitors, as well as with chemotherapy and radiation, and other novel approaches. Additionally, many promising combinations therapy trials involving ICIs are currently recruiting participants or are actively underway. Among them, certain studies garner considerable attention. In this section, we summarize the progress of single ICIs of PD-1, PD-L1, and CTLA-4 in CC clinical trials, as well as the research progress of some combinations of immune checkpoint inhibitors. The following table summarizes the results of completed clinical trials evaluating ICIs in the treatment of CC (**Table 1**).

**Table 1:** **Completed clinical trials with ICIs**.

Type	Drug	Trials	Phase	Treatment	Primary Endpoint	Results	AEs	Ref.
**Anti-PD-1**	Pembrolizumab	NCT02054806(KEYNOTE -028)	Ib	Pembrolizumab 10 mg/kg q2w for up to 2 years	ORR	17%	Rash and pyrexia	(25)
		NCT02628067(KEYNOTE-158)	II	Pembrolizumab 200 mg q3w for up to 2 years	ORR	12. 2%	Hypothyroidism, decreased appetite, fatigue, diarrhea	(26)
		NCT03444376	II	Pembrolizumab 200 mg q3w for up to 2 years + GX-188E (vaccine) 2 mg at week 1, 2, 4, 7, 13, 19	ORR	42%	Increased aspartate aminotransferase, syncope, pericardial effusion, hyperkalaemia, increased alanine aminotransferase	(27)
		NCT03635567(KEYNOTE-826)	III	Pembrolizumab (200 mg) or placebo every 3 weeks for up to 35 cycles plus platinum-based chemotherapy and, per investigator discretion, bevacizumab	PFS	10. 4mo	Anemia, nausea, diarrhea, peripheral neuropathy	(28)
		NCT02635360	II	Pembrolizumab 200 mg q3w for 3 cycles during vs, after CRT	PFS	4.8mo	Nausea, anemia, decreased lymphocyte count, vomiting, lymphocytopenia, leucopenia	(29)
	Nivolumab	NCT02257528(NRG-GY002)	II	Nivolumab 3 mg/kg q2w for up to 46 doses	ORR	4%	Blood and lymphatic system disorder, cardiac disorders	(30)
		NCT02488759(CheckMate 358)	II	Nivolumab 240 mg q2w for up to 2 years	ORR	26. 3%	Diarrhea, fatigue, pneumonitis, abdominal pain	(31)
	Cemiplimab	NCT03257267	III	Cemiplimab 350 mg q3w	PFS	12mo	Diarrhea, fatigue, and hypokalemia	(32)
		NCT02383212	I	Cemiplimab 3 mg/kg q2w for up to 48 weeks + hfRT during week 2	ORR	10%	Arthralgia, hypothyroidism, and maculopapular rash	(33)
	Balstilimab	NCT03104699	II	Balstilimab 3 mg/kg q2w for up to 24 months	ORR	15%	Immune-mediated enterocolitis, diarrhea	(34)
		NCT03495882	II	Balstilimab 3 mg/kg q2w and zalifrelimab 1 mg/kg q6w for up to 24 months	ORR	25.6%	Hypothyroidism, hyperthyroidism	(35)
**Anti-PD-L1**	Atezolizumab	NCT02921269	II	Atezolizumab 1200 mg q3w + bevacizumab 15 mg/kg q3w	ORR	0%	Hypertension, diarrhea, nausea, gastrointestinal	(36)
**Anti-CTLA4**	Ipilimumab	/	1/2	Ipilimumab 3 mg/kg, intravenously every 21 days for 4 cycles	PFS	2.5mo	Diarrhea, colitis	(37)

ORR, objective response rate; PFS, Progression free survival; AEs, adverse events.

#### 2.3.1 Anti-PD-1 therapy

To our knowledge, four early phase clinical trials have been published testing PD-1 ICIs in the treatment of CC, pembrolizumab, nivolumab, cemiplimab and balstilimab.

Pembrolizumab(MK-3475) is a highly selective, durable antitumor agent that inhibits the progression of advanced CC (38). A number of studies have focused on the improvement roles of pembrolizumab (39). In early 2017, a multicohort phase I study (KEYNOTE-028) evaluated the safety and efficacy of pembrolizumab in a cohort of PD-1-positive advanced solid tumors of 24 patients. Patients received 10 mg/kg pembrolizumab every 2 weeks for up to 24 months. The results showed that pembrolizumab was well tolerated and had durable antitumor activity in patients with PD-L1-positive advanced CC (25). Then, the safety and clinical benefits of pembrolizumab in advanced CC were subsequently investigated in the open-label, phase II, multicohort KEYCASE-158 trial (NCT02628067) (26). The interim results showed that the majority of patients (83. 7%) had PD-L1-positive tumors, with a higher objective response rate (ORR) in patients with PD-L1–positive tumors relative to the overall population (14. 6% vs 12. 2%, respectively). In particular, the ORR was 12.2% (95% CI, 6.5% to 20.4%), with 3 complete responses (CR) and 9 partial responses (PR). A total of 14.3% of these responses (95% CI, 7.4% to 24.1%) occurred in patients who had received one or more chemotherapies for recurrent or metastatic disease. This demonstrated the antitumor activity of pembrolizumab in patients with PD-1–positive advanced CC. Based on these encouraging results, pembrolizumab was approved by the Food and Drug Administration (FDA) in June 2018 for the treatment of patients with recurrent or metastatic CC who express PD-L1 post chemotherapy. Additionally, clinical trials of its combination therapy are also underway. A study of chemoradiotherapy with or without pembrolizumab for the treatment of locally advanced CC (NCT04221945) is recruiting, whose objective is to evaluate the efficacy and safety of pembrolizumab plus CCRT compared with placebo plus CCRT in participants with locally advanced CC (40). We look forward to the results of these studies to help guide clinical treatment.

Nivolumab, a complete PD-1 blocking antibody, was approved by the FDA in 2014 for use in melanoma patients and has attracted many clinical trials for the treatment of CC (41, 42). In the NRG-GY002 study, 26 enrolled patients with persistent/recurrent CC received nivolumab intravenously at 3 mg/kg every two weeks, followed by an additional 42 doses at 3 mg/kg every 2 weeks for a maximum of 46 doses until disease progression or adverse reactions prohibited treatment. The results showed a median follow-up for survival status of 32 months (range, 2-41.5 months), the median duration of SD was 5.7 months (range, 3.5-12.7 months), and the estimated PFS and OS at 6 months were 16% and 78, respectively (30). However, the limitation is that no significant correlation was found between PD-L1 expression and objective tumor response to nivolumab. In comparison, the results reported from the CheckMate 358 study showed that the ORR was 26.3% (95% CI, 9.1 to 51.2), and the median overall survival was 21. 9 months (95% CI, 15.1 months to not reached) among patients with CC (31). While the results reported here are of strong clinical interest, it should be noted that patients enrolled in the study were limited, and we still need large samples of experiments to confirm.

Cemiplimab is a fully human monoclonal antibody that targets PD-1 on T cells to inhibit T-cell activation (43). Cemiplimab is approved for the treatment of adult patients with metastatic or locally advanced cutaneous squamous cell carcinoma (CSCC) who are ineligible for radical surgery or radical radiation therapy (44, 45). A nonrandomized phase I expansion cohort study explored the safety and tolerability of cemiplimab as monotherapy or in combination with hfRT in patients in recurrent or metastatic CC. The results showed that cemiplimab treatment induced a response and clinical benefit in patients with recurrent or metastatic CC and that increasing hfRT after cemiplimab treatment did not significantly improve the ORR (33). Then, cemiplimab was quickly evaluated in a phase III randomized trial (NCT03257267) in which patients with disease progression after first-line platinum-containing chemotherapy were enrolled regardless of their PD-L1 status. Patients were randomly assigned in a 1:1 ratio to receive cemiplimab or investigator-chosen chemotherapy as control therapy, and for those randomized to cemiplimab, it was administered intravenously at a fixed dose of 350 mg every 21 days for up to 96 weeks. In the overall population, PFS was longer with cemiplimab than with chemotherapy (95% CI, 0.63 to 0.89). Objective responses occurred in 16.4% (95% CI, 12.5 to 21.1) of patients in the cemiplimab group compared with 6.3% (95% CI, 3.8 to 9.6) in the chemotherapy group. This is an exciting result that survival was significantly longer with cemiplimab than with single-agent chemotherapy among patients with recurrent CC after first-line platinum-containing chemotherapy (32).

Balstilimab (AGEN2034), as a PD-1 antagonist, is a fully human monoclonal antibody under investigation that enhances T-cell receptor (TCR) signaling and T-cell responsiveness under TCR stimulation conditions (46). A phase II clinical trial (NCT03104699) enrolled 161 women who were diagnosed with recurrent and/or metastatic CC and had relapsed after a prior platinum-based treatment regimen for advanced disease. The results showed the durable clinical activity of balstilimab (34). Patients with PD-L1-positive tumors had an ORR of 20%, while patients with PD-L1-negative tumors also responded to balstilimab (ORR, 7.9%). Another study of combination therapy with balstilimab has potential broad clinical implications for this disease. In an open-label phase II study (NCT03495882), the efficiency of the dual PD-1 and CTLA-4 checkpoint blockade inhibitors balstilimab and zalifrelimab was evaluated in patients with recurrent and/or metastatic CC. Approximately 155 patients were intravenously dosed with balstilimab 3 mg/kg once every 2 weeks and zalifrelimab 1 mg/kg every 6 weeks for up to 24 months. The results showed that the median follow-up was 21 months, and the confirmed ORR was 25.6% (95% CI, 18.8 to 33.9). Considering the expression of PD-L1, the ORRs were 32.8% and 9.1% in patients with PD-L1–positive and PD-L1–negative tumors, respectively (35).

#### 2.3.2 Anti-PD-L1 therapy

Three PD-L1 ICIs have been reported, atezolizumab, durvalumab, and avelumab, including at least six clinical trials evaluating their possible role in combination with other monoclonal antibodies or chemotherapy drugs in advanced, metastatic, persistent or recurrent CC.

Atezolizumab is a fully humanized drug that targets the PD-1/PD-L1 pathway by blocking PD-L1 ligand and has been widely used in immunotherapies for patients with extensive-stage small cell lung cancer (ES-SCLC), unresectable hepatocellular carcinoma and breast cancer (47). A phase II study of atezolizumab in combination with the vascular endothelial growth factor (VEGF) inhibitor bevacizumab in patients with advanced cc indicated that the addition of bevacizumab to PD-L1 blockade did not appear to increase the ORR (0%) in cervical cancer. Ten patients with advanced CC received bevacizumab 15 mg/kg intravenously and atezolizumab 1200 mg intravenously every 3 weeks, with a median PFS of 2.9 months (95% CI, 1.8 to 6) and a median OS of 8.9 months (95% CI, 3.4 to 21.9) (36). Fortunately, there is also an ongoing phase III clinical trial of platinum chemotherapy plus paclitaxel with bevacizumab and atezolizumab versus platinum chemotherapy plus paclitaxel and bevacizumab (48). We are looking forward to some new therapies for atezolizumab.

Another PD-L1 ICI, durvalumab, is an effective first-line treatment and an accepted standard of care option for patients with extensive-stage small cell lung cancer. Several studies on durvalumab are currently underway. An ongoing phase III randomized double-blind multicenter prospective placebo-controlled randomized trial was designed to investigate the efficacy of durvalumab in locally advanced CC. Approximately 714 patients would be randomized 1:1 to receive either durvalumab plus concurrent chemoradiotherapy (CCRT) or placebo plus CCRT (49). Another phase Ib study has been approved to evaluate the safety and tolerability of durvalumab combined with carbon-ion radiotherapy and weekly cisplatin in the treatment of locally advanced CC (50). The safety and efficacy of this strategy will be evaluated over a longer period of the study, and future phase II studies with additional patients are anticipated.

The efficacy of avelumab for treating patients with CC is being assessed in many clinical trials. Avelumab could kill cancer cells through antibody-dependent cell-mediated cytotoxicity (ADCC) and T-cell immune reactivity. It was approved by the U.S. FDA in a phase I/II study for the treatment of metastatic Merkel cell carcinoma and metastatic urothelial carcinoma (51, 52). There are two ongoing clinical trials of avelumab for the treatment of CC. One is a phase Ib/II study, avelumab in patients with recurrent or metastatic HPV-16 positive advanced malignancies aimed to evaluate the safety and tolerability of the combination of TG4001 (NCT03260023) (53). The other is a study evaluating avelumab in combination with axitinib in patients with persistent or recurrent CC following platinum-based chemotherapy that is recruiting patients worldwide, attracting considerable attentions (54). Fortunately, Bin Zhao and coworkers summarized 21 prospective trials with approximately 4000 subjects and revealed that avelumab monotherapy presents active antitumor activity (55). Compared with conventional treatment, avelumab monotherapy was associated with more tumor responses and fewer adverse events, and the pooled ORR was 14.18% (95% CI, 10.68%-18.08%). In particular, more PD-L1-positive patients than PD-L1-negative patients responded to avelumab monotherapy. In conclusion, the study strongly demonstrated that avelumab monotherapy had positive antitumor activity and did not show signs of increased toxicity. These findings provide a reasonable basis for the further application of avelumab in cancer treatment.

#### 2.3.3 Anti-CTLA-4 therapy

CTLA-4 is expressed on the surface of naive effector and regulatory T cells, and stimulation of naive T cells induces CTLA-4 upregulation and competition for B7 with CD28, resulting in T-cell activity suppression (56). While CTLA-4 and CD28 share the same ligand, CTLA-4 has a significantly stronger binding affinity and preferentially binds to CD80/CD86, resulting in immune response inactivation. Therefore, blockade of the CTLA-4 antigen increases antigen presentation to the immune system, resulting in an expanded killer T-cell response to antigens.

Many studies have focused on the safety and efficiency of anti-CTLA4 therapy. Ipilimumab is the most studied anti-CTLA-4 therapy in patients with CC. In a phase 1/2 trial, 42 patients with metastatic CC received intravenous ipilimumab therapy. The response rate was 3% in 34 evaluable patients, PFS was 2.5 months (95% CI, 2.1-3.2), and OS was 8.5 months (95% CI, 3.6 months not reached) (37). Additionally, a phase I clinical trial (GOG-9929) corroborated these findings. The data suggested that CRT alone and combined with ipilimumab immunotherapy show immunomodulatory activity with locally advanced CC (57).

#### 2.3.4 Immune-related adverse events of ICIs

ICI therapies have made great strides and have improved overall survival in advanced and recurrent/metastatic CC. However, these therapies have led to a particular group of side effects called irAEs. ICIs work by releasing T cells from the immune system that fight tumor cells, and the disadvantage of an augmented immune response driven by T-cell activation is the potential autoimmune-related inflammation of normal tissues. The irAEs typically originate in the skin, gastrointestinal tract, liver, and endocrine system. The most frequent endocrine irAEs associated with anti-PD-1 mAb treatment are thyroid dysfunctions, and other related irAEs include diarrhea, colitis, hepatitis, skin toxicities and endocrinopathies. Hypophysitis is mostly linked to anti-CTLA-4 treatment, diarrhea, colitis and liver dysfunction (58). Despite tumor heterogeneity, the treatment of irAEs is still mainly glucocorticoid therapy. Most symptomatic irAEs, except for endocrine disease, are controlled with several weeks of glucocorticoid therapy and respond well. Some irAEs could become chronic and require lifelong treatment, such as hormone supplementation or immunosuppression (59).

## 3 Microbial infections and ICIs

### 3.1 Vaginal microecology and HPV infection

In the female reproductive tract, vaginal microecology is composed of vaginal anatomy, microbial flora, local immune system, estrogen and other endocrine regulation. The symbiotic microbial community structure and estrogen dynamic changes play a leading role in maintaining vaginal microecological homeostasis effects (60). Caselli’s prospective study showed that vaginal microecological disturbances were associated with HPV infection and cervical HSIL progression (61). When the vaginal microenvironment is destroyed, the immune cells are dysfunctional, and the autoimmune barrier and local immune function in the reproductive tract are destroyed, increasing the susceptibility to HPV and reducing the natural elimination rate of HPV. At present, it is believed that the reduction of cellular immune barrier function and the inhibition of local immune response are the mechanisms by which vaginal microecology leads to cervical lesions (62). In addition, the imbalance of vaginal microecology leads to the destruction of the vaginal innate immune barrier, increases the adhesion of HPV virus, activates inflammatory transcription factors, stimulates the continuous activation of various inflammatory factors, induces or aggravates cervical tissue damage, and further increases the carcinogenicity of HPV. After infection, the diversity of vaginal microflora and changes in the local microenvironment of the cervix destroy the host’s immune defense mechanism, which causes the expression of inflammatory factors and leads to vaginal flora disorder (63, 64).

The relationship between vaginal microecological disorder and HPV infection and immune disorder of CC leads us to hypothesize. First, in HPV-positive advanced/recurrent CC patients, we generally perform systemic surgery, radiotherapy, chemotherapy and targeted therapy. At the same time, we could maintain the balance of vaginal microecology, increase the colonization of dominant bacilli, locally increase the stability of the microenvironment in the cervical region, improve immunity and prevent vaginal inflammation such as AV, BV, TV, etc., which could increase the effect of systemic treatment and improve patients’ quality of life. Second, it should be considered whether the persistent HPV positivity after surgery is related to vaginal microecological disorders, especially for patients with reduced hormone levels after surgery and adjuvant therapy. In clinical treatment, vaginal supplementation with probiotics, rebuilding the balance of vaginal microbiota and improving the local immune barrier could speed up the conversion of HPV to negative. Finally, we emphasize the research on vaginal microbial imbalance and related metabolic markers to provide new ideas for CC prevention and early screening.

### 3.2 Intestinal microenvironment and ICIs

Gut microbiota homeostasis is inextricably linked to the development and maturation of the immune system (65). Gut microbiota imbalance is an important driver of cancer immune system dysregulation. A gut microbiota in a state of symbiotic balance would facilitate antitumor cell activity, improve resistance to immunosuppressive therapy and alleviate the side effects of antitumor therapy.

The influence of the gut microbiota on the response to immune checkpoint inhibitor therapy in CC has not been explored, but this could be extrapolated from studies in other cancers. Adoptive T-cell therapy (ACT) in a mouse model of CC is influenced by the gut microbiota. Fecal analysis of mice that responded well to ACT revealed different bacteroides taxa, including the bacteroidetes, parabacteroides, prevotella, rikenellaceae families and the candidate bacteroidetes S24-7 family. The mice with poor efficacy were dominated by bacteroidetes S24-7, and no other bacteroidetes were detected (66). A single-arm clinical trial found that FMT (fecal microbiota transplantation) and anti-PD-1 therapy altered the PD-1 response in selected patients with advanced melanoma resistance by altering the gut microbiota and reprogramming the tumor microenvironment (67). In addition, in a group of controlled studies, patients with non-small cell lung cancer, renal cell carcinoma or urothelial carcinoma who received antibiotics before and after treatment with immunosuppressants had significantly higher rates of PFS and overall survival than those who did not receive antibiotics. This suggests that disrupting the gut microbiota (through antibiotic use) may affect or even disrupt the therapeutic efficacy of immunosuppressants (68). It has been reported that oral administration of bifidobacteria in tumor-bearing mice can promote the therapeutic effect of immunosuppressive agents during antitumor therapy with immunosuppressive agents (69).

Obviously, there are increasing evidences showing that the regulation of the gut microbiota of tumor patients may become a breakthrough point in tumor immunotherapy. Although the gut microbiota has been found to play an important role in some cancers and animal models, it is unknown whether this can be extrapolated to the treatment of CC patients. For CC patients, studying their gut microflora or underlying molecular mechanisms would help to develop new models of CC treatment to enhance the efficacy of CC immunotherapy.

## 4 Conclusion

Cervical cancer (CC) is the 4th leading cause of cancer deaths in women worldwide. Surgery, chemotherapy, radiotherapy and chemoradiation therapy are conventional treatments for CC. However, the treatment options are limited, and the prognosis is poor for patients with advanced and recurrent/metastatic CC, with a 5-year survival rate of only 16 to 58% in advanced stages. In recent years, ICIs therapies have shown promise in greatly improving overall survival and quality of life in advanced and recurrent/metastatic CC. Many clinical trials of ICIs monotherapies or combinations are now showing great potential. Combinations of immunotherapy, chemotherapy, and radiotherapy with ICIs have been shown to yield more potential antitumor effects in clinical trials. However, compared with other types of cancer, there are still very few phase III trials for CC. For clinical trials that have been completed, these characteristics provided justification for further evaluating its efficacy in cancer treatment. Further studies are needed to validate these findings in real-world practice. For clinical trials that are ongoing, we look forward to the results of these immunotherapy clinical trials and believe that the new immunotherapy targets will also bring hope to the treatment of patients with advanced and metastatic CC.

The different levels of immune-related adverse events are an emerging challenge for ICIs therapy. Hopefully, we find that vaginal microbial dysbiosis is closely related to HPV infection, and vaginal infection caused by microorganisms could lead to damage to the mucous membranes of the genital tract, thereby increasing the risk of HPV invasion. In addition, the effect of the intestinal microbiome on the immune system promotes the recurrence and metastasis of CC. With an improved understanding of the effects of ICIs and microbial infection on immune function, we would be able to optimally deliver therapy to patients with advanced and recurrent/metastatic CC. Finally, an arsenal of conventional anticancer therapies combined with multiple and novel ICIs, and together with interventions on the microbiota will be able to increase the fraction of oncological patients that experience complete and durable remissions at the cost of manageable side effects.

## Author contributions

XH drafted the manuscript and finished the figures, WC assisted in the processing of data, and XX provided feedback and guidance. All authors contributed to the article and approved the submitted version.

## Funding

This project was supported by grants from the excellent and top-notch talent cultivation project in colleges and universities in Anhui Province(gxgnfx2022039); Talents Program for Academic Leaders and Reserve Candidates of Wannan Medical College (No. School Administration Letter (2021) No. 46).

## Conflict of interest

The authors declare that the research was conducted in the absence of any commercial or financial relationships that could be construed as a potential conflict of interest.

## Publisher’s note

All claims expressed in this article are solely those of the authors and do not necessarily represent those of their affiliated organizations, or those of the publisher, the editors and the reviewers. Any product that may be evaluated in this article, or claim that may be made by its manufacturer, is not guaranteed or endorsed by the publisher.
